# Phase Behavior and Percolation of a Primitive Model of Laponite Suspension: Wertheim’s Thermodynamic Perturbation Theory with Anisotropic Reference Particles

**DOI:** 10.1021/acsomega.5c12221

**Published:** 2026-03-23

**Authors:** Yurij V. Kalyuzhnyi

**Affiliations:** † Faculty of Chemistry and Chemical Technology, University of Ljubljana, Večna pot 113, 1000 Ljubljana, Slovenia; ‡ Yukhnovskii Institute for Condensed Matter Physics, Svientsitskoho 1, 79011 Lviv, Ukraine

## Abstract

The computation of the properties of associative fluids within Wertheim’s multidensity thermodynamic perturbation theory becomes particularly challenging when the reference system is composed of strongly anisotropic, nonspherical particles. We develop a simple and efficient framework that combines thermodynamic perturbation theory with the interaction site model formalism of Chandler and Andersen. The method enables an accurate treatment of associating fluids with anisotropic reference particles and is illustrated through calculations of the phase behavior and percolation properties of the primitive model of Laponite suspensions.

## Graphical abstract

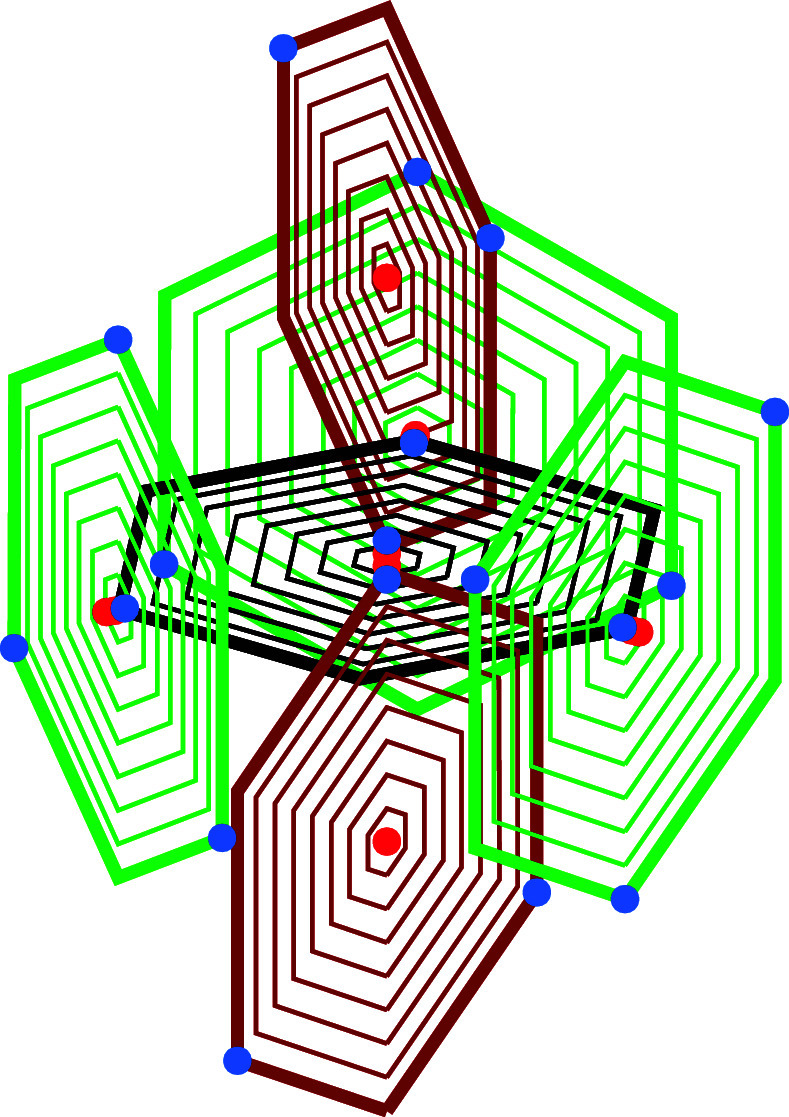

## Introduction

Since Wertheim’s pioneering work,^1–5^ substantial progress has been achieved in the development and application of multidensity thermodynamic perturbation theory (TPT) for associative fluids. This framework and its extensions have been successfully employed to describe the properties of fluids composed of small associative molecules and their mixtures, as well as polymers, liquid crystals, surfactants, colloids, and biological macromolecules (including proteins).^6–16^ A common feature of nearly all these studies is the assumption that the particles in the corresponding reference systems are spherical. However, recent advances in the synthesis of colloidal building blocks with diverse shapes and functionalities^17^ make the theoretical description of their self-assembly behavior increasingly significant.

Application of multidensity thermodynamic perturbation theory (TPT) requires knowledge of the structural and thermodynamic properties of the corresponding reference system, i.e., the system in which the interactions responsible for bonding (association) are switched off. In particular, the pair distribution function of the reference system is required to calculate the fractions of particles in a given bonding state using a statistical-mechanical analogue of the law of mass action. The key quantity entering this relation is an integral of the product of two functions over the entire phase space: the pair distribution function and the Mayer function of the associative potential. The latter typically arises from off-center square-well sites located on particle surfaces. For particles with complex and highly nonspherical shapes, both functions exhibit strong orientational dependence, making the evaluation of the corresponding integrals a formidable task. Moreover, the calculation of the angular-dependent pair distribution function itself is highly nontrivial. These difficulties likely constitute the main obstacle to the widespread application of Wertheim’s TPT to fluids composed of particles with complex shapes.

In this paper, we propose a simple and efficient scheme that enables the application of Wertheim’s TPT to associating fluids composed of highly nonspherical particles. The approach combines multidensity TPT with the interaction-site model formalism of Chandler and Andersen^18^ which is employed to describe the properties of the reference system. The scheme is illustrated through its application to the study of the phase behavior of the primitive model of Laponite. In the Supporting Information we present the version of our approach formulated for a fluid of spherical particles.

## Primitive Model of Laponite

The primitive model of a Laponite nanoparticle is represented by a collection of 19 hard spheres of size σ arranged to form a hexagonal-shaped platelike particle, with their nearest neighbors in contact. In addition, three symmetrically located hard spheres at the corners of the hexagon are decorated with one square-well site of type *B* each, and the central hard sphere of the hexagon is decorated with two square-well sites of type *A*, placed on its two opposite faces (see Figure 1). The number of sites of type *A* is *n*_*A*_ = 2 and of type *B* is *n*_*B*_ = 3. Site–site square-well interaction is acting only between sites of different type and these sites are placed on the surface of the respective hard sphere. We identify four groups of equivalent hard-sphere sites of the model, which we denote as *C*, *D*, *E* and *F* (see Figure 1). Each group of type *K* includes *n*_*K*_ sites denoted as *K*_1_, *K*_2_, , *K*_*n*_*K*__. For this model *n*_*C*_ = 1 and *n*_*D*_ = *n*_*E*_ = *n*_*F*_ = 6.

**Figure 1:**
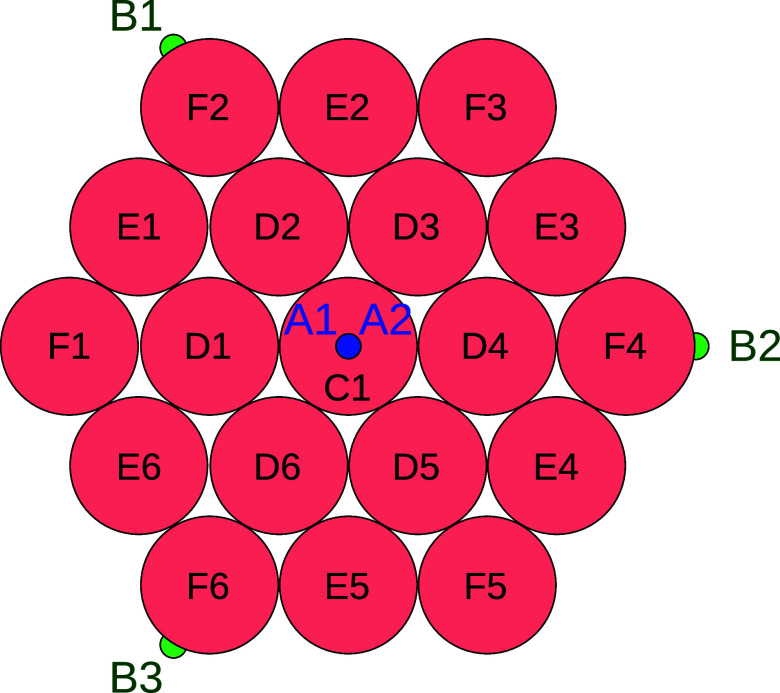
Schematic representation of the primitive model of Laponite nanoparticles. Here square-well sites are denoted as *A* (blue) and *B* (green) and hard-sphere sites are denoted as *C*, *D*, *E*, *F* (black).

The corresponding interparticle pair potential is

1 $$U\left(12\right)=U_{ref}\left(12\right)+\sum_{i=1}^{n_{A}}\sum_{j=1}^{n_{B}}\left[U_{A_{i}B_{j}}^{\left(\text{as}\right)}\left(12\right)+U_{B_{j}A_{i}}^{\left(\text{as}\right)}\left(12\right)\right]$$

where *U*_ref_(12) is interparticle pair potential of the reference system

2 $$U_{ref}\left(12\right)=\sum_{KL=C}^{F}\sum_{i=1}^{n_{K}}\sum_{j=1}^{n_{L}}U_{K_{i}L_{j}}^{\left(hs\right)}\left(12\right)$$

$U_{A_{i}B_{j}}^{\left(\text{as}\right)}\left(12\right)$ is the site–site square-well potential acting between site *A*_*i*_ of particle 1 and site *B*_*j*_ of particle 2, i.e.

3 $$U_{A_{i}B_{j}}\left(12\right)=U_{A_{i}B_{j}}\left(z_{12}\right)=\{\begin{array}{ll} -\epsilon_{AB}, & z_{12}<\delta \\ 0, & z_{12}>\delta \end{array}$$

*z*_12_ is the distance between sites *A*_*i*_ and *B*_*j*_, ϵ_*AB*_ is the depth of the square-well potential, $U_{K_{i}L_{j}}^{\left(hs\right)}\left(12\right)$ is the site–site hard-sphere potential acting between the site *K*_*i*_ of the particle 1 and site *L*_*j*_ of the particle 2, i.e.

4 $$U_{K_{i}L_{j}}^{\left(hs\right)}\left(12\right)=U_{K_{i}L_{j}}^{\left(hs\right)}\left(d_{12}\right)=\{\begin{array}{ll} \infty , & d_{12}<\sigma \\ 0, & d_{12}>\sigma \end{array}$$

*d*_12_ is the distance between sites *K*_*i*_ and *L*_*j*_. Here *K* and *L* take the values *C*, *D*, *F*. The width of the square-well site–site potential δ was chosen to satisfy the “one bond per site” condition $\delta <\frac{1}{2}\sigma \left[\sqrt{5-2\sqrt{3}}-1\right]$ , i.e. each site of one particle can be involved in a bond with only one site of another particle.

## Theory

The first-order version of TPT (TPT1) is formulated in terms of the Helmholtz free energy *A* of the model, which is represented as the sum of two terms, i.e.

5 $$A=A_{ref}+\Delta A_{\text{as}}$$

where *A*_ref_ is Helmholtz free energy of the reference system and Δ*A*_as_ is the contribution to Helmholtz free energy due to association. Taking into account the symmetry of the model, we have^5,19^

6 $$\frac{\beta \Delta A_{\text{as}}}{V}=\rho \left[\ln\left(X_{A}^{2}X_{B}^{3}\right)-\frac{1}{2}\left(2X_{A}+3X_{B}\right)+\frac{5}{2}\right]$$

where β = 1/(*k*_B_ *T*), *k*_B_ is Boltzmann’s constant, *T* is the temperature, ρ is the number density, *V* is the volume of the system, *X*_*K*_ ≡ *X*_*K*_*i*__ (*K* = *A*, *B*) denotes the fraction of particles whose attractive site of type K is not bonded. These fractions follow from the solution of the “mass action law” equation,^5,19^ i.e.

7 $$X_{A}=\frac{-1-\rho I_{AB}+\sqrt{{\left(1+\rho I_{AB}\right)}^{2}+8\rho I_{AB}}}{2\rho I_{AB}}$$

and

8 $$X_{B}=\frac{1}{3}\left(1+2X_{A}\right)$$

where

9 $$I_{AB}=\int {\left\langle g_{ref}\left(12\right)f_{AB}\left(12\right)\right\rangle }_{\Omega_{1}\Omega_{2}}dr_{12}$$

here *g*_ref_(12) is the pair distribution function of the reference system, *f*_*AB*_^(as)^(12) is the Mayer function for the site–site square-well potential, i.e *f*_*AB*_^(as)^(12) = exp­[−β*U*_*AB*_^(as)^(12)] – 1, and ${\left\langle ...\right\rangle }_{\Omega_{1}\Omega_{2}}$ denotes angular averaging with respect to the orientations of particles 1 and 2. This integral can be evaluated by choosing an arbitrary location for the origin of the coordinate system associated with each particle. If the origin is placed at the position of the corresponding attractive site of the particle (site *A* of particle 1 and site *B* of particle 2), we obtain

10 $$I_{AB}=4\pi \int {\left\langle g_{ref}\left(12\right)\right\rangle }_{\Omega_{1}\Omega_{2}}r_{12}^{2}f_{AB}\left(r_{12}\right)dr_{12}=4\pi \left(e^{-\beta \epsilon_{AB}}-1\right)\int_{0}^{\delta }r_{12}^{2}g_{AB}^{\left(ref\right)}\left(r_{12}\right)dr_{12}$$

where *r*_12_ is the distance between sites *A* and *B* of particles 1 and 2, respectively, and ${\left\langle g_{ref}\left(12\right)\right\rangle }_{\Omega_{1}\Omega_{2}}=g_{AB}^{\left(ref\right)}\left(r_{12}\right)$ is the site–site pair distribution function between two auxiliary sites *A* and *B* of the reference system.^20,21^ This correlation function can be calculated using the reference interaction site model (RISM) approach due to Chandler.^18,22^

All other thermodynamic properties follow from standard thermodynamical relations. For the pressure *P* and the chemical potential μ we have

11 $$\beta P=\rho +\beta P_{ref}^{\left(ex\right)}+\beta \Delta P_{\text{as}}$$

12 $$\beta \mu =\ln\left(\rho \Lambda^{3}\right)+\beta \mu_{ref}^{\left(ex\right)}+\beta \Delta \mu_{\text{as}}$$

where *P*_ref_^(ex)^ and μ_ref_^(ex)^ are excess pressure and chemical potential potential, respectively. For the contributions to chemical potential Δμ_as_ and pressure Δ*P*_as_ due to association we have

13 $$\Delta \mu_{\text{as}}={\left(\frac{\partial \left(\Delta A_{\text{as}}/V\right)}{\partial \rho }\right)}_{T,V}$$

14 $$\Delta P_{\text{as}}=\rho \Delta \mu_{\text{as}}-\Delta A_{\text{as}}/V$$

Note that these expressions for the pressure [eq 11] and the chemical potential [eq 12] are thermodynamically consistent, as they satisfy the Gibbs–Duhem relation.

## Properties of the Reference System

The reference system is represented by a fluid of Laponite particles with zero site–site square-well potential depth, i.e. ϵ_*AB*_ = 0. Models of this type have previously been studied using the site–site Ornstein–Zernike (SSOZ) equation combined with closure relations similar to those employed in the integral equation theory of simple fluids. It is generally accepted that while the hypernetted-chain (HNC) and mean spherical approximations (MSA) are effective for systems with long–range interactions, the Percus–Yevick (PY) approximation provides more accurate results for systems dominated by short-range interactions.^22,23^ Recently, the SSOZ equation supplemented by the PY closure was successfully applied to investigate the properties of a Laponite model closely related to the one adopted here for our reference system.^24^ Following this earlier work, we compute both the thermodynamic and structural properties of the reference system using the appropriate form of the SSOZ equation combined with a PY-like closure, which has been shown to provide a reliable description of short-range correlations in such systems. The thermodynamics of the reference system does not depend on the presence or absence of auxiliary sites, therefore we consider the model with hard-sphere sites only. There are 19 hard-sphere sites, thus the dimension of the matrices representing site–site correlation functions in the SSOZ equation is 19 × 19. However, taking into account the symmetry of the model, the dimensionality of the SSOZ equation can be reduced. We follow here the scheme proposed by Raineri and Stell^25^ and recently used by Costa et al.^24^ to study a model similar to the current one. This scheme does not involve any preaveraging steps and therefore introduces no additional approximations. The reduced version of the SSOZ equation for the model at hand is

15 ĥ(k)=Ŵ(k)Ĉ(k)Ŵ(k)+ρŴ(k)Ĉ(k)ĥ(k)

where Ŵ(k) and Ĉ(k) are matrices with elements

16 $${Ŵ}_{KL}\left(k\right)=\frac{1}{n_{L}}\sum_{j=1}^{n_{L}}{Ŝ}_{ij}\left(k\right)=\frac{1}{n_{K}}\sum_{i=1}^{n_{K}}{Ŝ}_{ij}\left(k\right)={Ŵ}_{LK}\left(k\right)$$

and ${Ĉ}_{KL}\left(k\right)=n_{K}{ĉ}_{ij}\left(k\right)n_{L}$ . The corresponding PY-like closure is

17 $$\{\begin{array}{ll} C_{KL}\left(r\right)=0, & r>\sigma \\ h_{KL}\left(r\right)=-1, & r\leq \sigma \end{array}$$

The solution of this set of equations is used to calculate the thermodynamic properties of the model using compressibility route.^24^

The inverse compressibility can be expressed in terms of the direct correlation functions as follows^22,26^

18 $$\beta {\left(\frac{\partial P_{ref}}{\partial \rho }\right)}_{T}=1-4\pi \rho \sum_{KL}\int r^{2}C_{KL}\left(r\right)dr$$

where *P*_ref_ denotes the pressure of the reference system. In addition, using the Gibbs–Duhem relation, one obtains^27^

19 $$\beta \mu_{ref}^{\left(ex\right)}=\left(\frac{\beta P_{ref}}{\rho }-1\right)+\int_{0}^{\rho }\frac{1}{\rho^{\prime }}\left(\frac{\beta P_{ref}}{\rho^{\prime }}-1\right)$$

where μ_ref_^(ex)^ is the excess chemical potential of the reference system. Combining eqs 18 and 19, we obtain explicit expressions for the excess pressure *P*_ref_^(ex)^ and the chemical potential μ_ref_^(ex)^

20 $$\beta P_{ref}^{\left(ex\right)}=-4\pi \int_{0}^{\rho }\rho^{\prime }d\rho^{\prime }\sum_{KL}\int r^{2}C_{KL}\left(r\right)dr$$

and

21 $$\beta \mu_{ref}^{\left(ex\right)}=-4\pi \int_{0}^{\rho }d\rho^{\prime }\sum_{KL}\int r^{2}C_{KL}\left(r\right)dr$$

The calculation of the structure properties requires the solution of the SSOZ equation formulated for a model that in addition to hard-sphere sites includes also auxiliary sites. We consider a model with eight auxiliary sites. Five of the sites represent square-well sites with ϵ_*AB*_ = 0, while the remaining three are introduced to increase the degree of symmetry of the model. The last three sites are placed on the surface of three rim hard-sphere sites that are not decorated with square-well sites (see Figure 1). Thus, there are six groups of equivalent sites, i.e. *A*, *B*, *C*, *D*, *E*, *F*, where the first two represent auxiliary sites and the last four represent hard-sphere sites. Here *n*_*A*_ = 2 and *n*_*B*_ = 6. Now the dimension of the matrices that enter the SSOZ eq 15 is 6 × 6. The solution of this version of SSOZ equation gives *g*_*AB*_^(ref)^(*r*), which is used to calculate the integral *I*_*AB*_ (10).

## Results and Discussion

Using the theory developed above, we calculate the liquid–gas phase diagram and the percolation threshold line of the primitive model of Laponite suspension. The densities of the coexisting phases follow from the solution of the set of equations representing the phase equilibrium conditions

22 $$\{\begin{array}{l} P\left(T,\rho_{g}\right)=P\left(T,\rho_{l}\right)\\ \mu \left(T,\rho_{g}\right)=\mu \left(T,\rho_{l}\right) \end{array}$$

where ρ_*g*_ and ρ_*l*_ are the densities of low-density and high-density phases, respectively. The percolation threshold line was calculated following the scheme suggested by Tavares et al.^28^ This scheme combines TPT of Wertheim and Flory–Stockmayer theory of percolation^29–31^ For a detailed description of the scheme, we refer readers to the original publications; here we present only the final set of equations to be solved. The threshold line points on the ρ vs *T* coordinate plane satisfy the following equation

23 $$\sqrt{T_{\Sigma }^{2}-4T_{\Pi }\left(\frac{1-X_{A}}{T_{A}}+\frac{1-X_{B}}{T_{B}}-\frac{1}{n_{\Pi }}\right)}+T_{\Sigma }-2=0$$

where $T_{L}=n_{L}\left(1-X_{L}\right)\prod_{K=A}^{B}q_{K}^{n_{K}-1}$ , *T*_Π_ = *T*_*A*_ *T*_*B*_, *n*_Π_ = *n*_*A*_ *n*_*B*_, *T*_Σ_ = *T*_*A*_ + *T*_*B*_ and *q*_*L*_ is obtained from the solution of the set of equations

24 $$X_{L}-\left[1-\left(1-X_{L}\right)\prod_{K=A}^{B}q_{K}^{n_{K}-1}\right]q_{L}=0$$

In Figure 2 we present the theoretical and Monte Carlo computer simulation results^32,33^ for the phase diagram and the percolation threshold line using T* vs ρ* coordinate frame. Here T* = *k*_B_ *T*/ϵ_*AB*_ and ρ* = ρσ^3^, where *k*_B_ is the Boltzmann constant. In general, the accuracy of the present implementation of Wertheim’s first-order TPT is comparable to that observed for hard-sphere systems with several off-center square-well sites,^34^ where theoretical predictions are only qualitatively accurate. While our theory provides relatively accurate estimates of the critical density ρ_c_^*^, the predicted critical temperature *T*_c_^*^, and consequently the percolation threshold line, are less accurate, with *T*_c_^*^ overestimated by about 9%. Furthermore, the liquid branch of the phase diagram at low temperatures is shifted toward higher densities, by approximately a factor of 1.7. Given that theoretical predictions are generally reliable only at a qualitative level, the reasonably good agreement obtained for the critical density may partly result from a fortuitous compensation of errors arising from different sources, such as inaccuracies in the RISM-based description of the reference system and the intrinsic limitations of TPT1 (see below), among others. The width of the coexistence region and the position of the liquid branch are primarily governed by the effective valency υ_eff_, i.e., the average number of bonds per particle in the limit of vanishing temperature.^33^ As υ_eff_ increases, the phase diagram broadens and its liquid branch moves to higher densities.^34^ Within TPT1 framework, the average number of bonds per particle, *m*_av_, is given by^3,4,35^

25 $$m_{av}=\sum_{m=1}^{5}m\sum_{m_{A}+m_{B}=m}x_{m_{A}m_{B}}$$

where $x_{m_{A}m_{B}}$ denotes the fraction of particles with *m*_*A*_ and *m*_*B*_ sites of the type *A* and *B* bonded, respectively

26 $$x_{m_{A}m_{B}}=\prod_{L=A}^{B}\frac{n_{L}!X_{L}^{\Delta n_{L}}{\left(1-X_{L}\right)}^{m_{L}}}{m_{L}!\left(\Delta n_{L}\right)!}$$

*m*_*A*_ = 0, 1, 2, *m*_*B*_ = 0, 1, 2, 3, and Δ*n*_*L*_ = *n*_*L*_ – *m*_*L*_. Here *n*_*L*_ is the total number of sites of the type *L*. According to 7 and 8 $\lim_{T^{*}\to 0}X_{A}=0$ and $\lim_{T^{*}\to 0}X_{B}=1/3$ . Thus, at infinity low temperature all sites of the type *A* are bonded. Using this result and expression for *m*_*av*_ [Disp-formula eq26] we have

27 $$\upsilon_{eff}^{\left(TPT\right)}=\lim_{T^{*}\to 0}m_{av}=\frac{14}{3}$$

**Figure 2:**
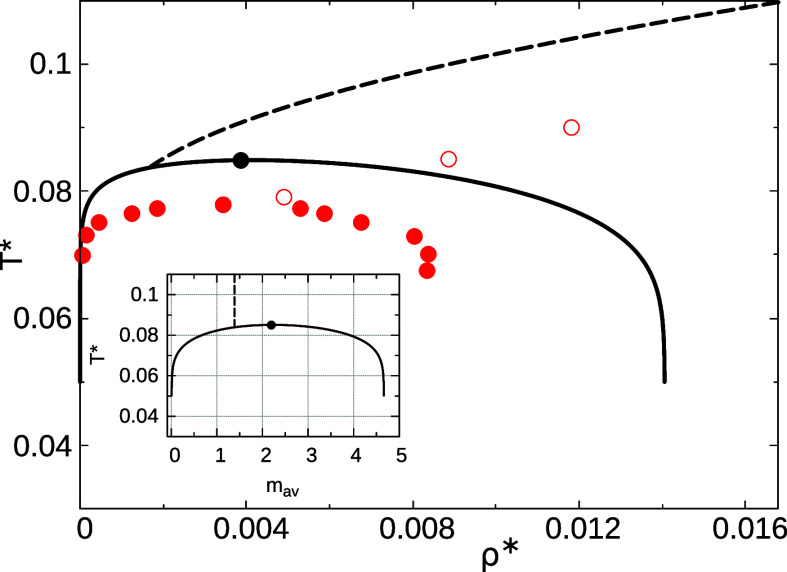
Phase diagram and percolation threshold line of the primitive model of a Laponite suspension in the T* vs ρ* plane and, in the inset, in the T* vs *m*_av_ representation. Solid and dashed curves show the theoretical binodals and percolation line, respectively, while filled black circles indicate the theoretical critical point. Red filled and open circles represent computer simulation data^32^ for the binodal and percolation threshold line, respectively. Reprinted in part with permission from ref 32. Copyright 2011 Royal Society of Chemistry.

This value of the effective valency determines the position of the liquid branch in the theoretical phase diagram at low temperatures. The inset of Figure 2 shows the temperature dependence of *m*_av_ along the coexistence line. It is seen that for temperatures below approximately T* ≈ 0.065, *m*_av_ varies only weakly and approaches its limiting value as the temperature decreases. However, according to MC simulations,^32,33^ the exact effective valency is lower, υ_eff_^(MC)^ = 4. Consequently, the liquid branch of the MC phase diagram appears at smaller densities than in the theoretical prediction. The fact that the exact value of the effective valency is smaller than υ_eff_^(TPT)^ suggests that $\lim_{T^{*}\to 0}X_{A}\neq 0$ , i.e., even in the zero-temperature limit a finite fraction of particles retains unbonded *A* sites. This behavior arises from blocking effects, where the bonding of one site prevents the formation of bonds at another site. Thus, the quantitative discrepancies between the theoretical and exact results for the liquid branch of the phase diagram primarily originate from the intrinsic limitations of TPT1 rather than from the proposed framework and are model-specific. TPT1 does not account for blocking effects,^5^ which become significant due to the highly anisotropic shape of the particles. For models with more moderate shape anisotropy and/or patch arrangements that minimize blocking effects, the accuracy of the present approach is expected to be comparable to that of the original TPT1 for spherical particles, provided that the reference system is described with a similar level of accuracy. In particular, for the models with hard-sphere reference system present approach coincide with original TPT1 as formulated by Wertheim (see Supporting Information to this article). In general, the predictions of our theory for the phase behavior of the present Laponite model should be regarded as qualitatively accurate only. Nevertheless, the overall conclusions of this study remain consistent with those obtained from Monte Carlo simulations, demonstrating that the proposed model qualitatively reproduces the formation of the empty-liquid state observed experimentally in Laponite suspensions.^32,33^

In summary, we have developed a theoretical framework that extends Wertheim’s multidensity thermodynamic perturbation theory to associative fluids with reference systems composed of strongly nonspherical, anisotropic particles. The approach combines TPT with the interaction site model (ISM) formalism for molecular fluids, where the ISM component is employed to describe the structure and thermodynamics of the reference system. We expect that this framework will stimulate further theoretical and computational studies on self-assembly and phase behavior in systems of anisotropic colloidal particles, opening a new avenue for advancing the theory of associating fluids.

## Supplementary Material



## Notes

Details of the formulation of the present approach for a fluid with spherical reference particles (PDF)
