# Supplementary material for: Phase Behavior and Percolation of a Primitive Model of Laponite Suspension: Wertheim’s Thermodynamic Perturbation Theory with Anisotropic Reference Particles
Source: ACS Omega. 2026 Mar 23;11(13):20600–4. doi: 10.1021/acsomega.5c12221 (PMC13063183; doi:10.1021/acsomega.5c12221)
Supplement: Supplementary file 1 [file ao5c12221_si_001.pdf]

Supporting information for: Phase Behavior and Percolation of a Primitive Model of Laponite Suspension: Wertheim's Thermodynamic Perturbation Theory with Anisotropic Reference Particles

Y. V. Kalyuzhnyi*

University of Ljubljana, Faculty of Chemistry and Chemical Technology, Večna pot 113, Ljubljana, Slovenia,

Yukhnovskii Institute for Condensed Matter Physics, Svientsitskoho 1, 79011 Lviv, Ukraine

E-mail: yukal@icmp.lviv.ua

*To whom correspondence should be addressed

Initially, TPT for associative fluids was formulated for a model represented by a fluid of hard spheres of size σ with n_s additional off-center square-well sites located at a distance $d \leq \sigma/2$ from the center of a hard sphere.[S1–S4] The corresponding interparticle pair potential is

$$U(12) = U_{hs}(r) + \sum_{KL} U_{KL}(12), \tag{1}$$

where $U_{hs}(r)$ is the hard-sphere potential, $U_{KL}(12)$ is a site-site square-well potential acting between the site K of particle 1 and the site L of particle 2, i.e.

$$U_{KL}(12) = U_{KL}(z_{12}) = \begin{cases} \epsilon_{KL}, & z_{12} < \delta \\ 0, & z_{12} > \delta \end{cases}, \tag{2}$$

z_{12} is the distance between sites K and L, i.e. $z_{12} = |\mathbf{r}_2 + \mathbf{d}_L(\Omega_1) - \mathbf{r}_1 - \mathbf{d}_K(\Omega_2)|$, $\mathbf{d}_K(\mathbf{d}_L)$ is a vector of length d connecting the center of the particle and its site $K(L)$, $1(2)$ denotes the position $\mathbf{r}_1(\mathbf{r}_2)$ and orientation $\Omega_1(\Omega_2)$ of particle $1(2)$. Here K and L take n_s values $A, B, C, \ldots$. The parameters of the square-well site-site potential d and δ were chosen to satisfy the 'one bond per site' condition $\delta < \sqrt{\sigma^2 + d^2 - \sigma d\sqrt{3}} - d$, i.e. each site of one particle can be involved in a bond with only one site of another particle. The first-order version of TPT (TPT1) is formulated in terms of the Helmholtz free energy A of the model, which is represented as the sum of two terms, i.e.

$$A = A_{ref} + \Delta A_{as}, \tag{3}$$

where A_{ref} is Helmholtz free energy of the reference system and ΔA_{as} is the contribution to the Helmholtz free energy due to association,

$$\beta \frac{\Delta A_{as}}{N} = \sum_K \left(\ln X_K - \frac{1}{2} X_K \right) + \frac{1}{2} n_s. \tag{4}$$

Here $\beta = 1/(k_B T)$, k_B is Boltzmann's constant, T is the temperature, N is the number of

the particles, X_K is the fraction of the particles with an attractive site of type K not bonded. This fraction follows from the solution of the set of equations

$$\rho X_K \sum_L X_L I_{KL} + X_K - 1 = 0, \tag{5}$$

where

$$I_{KL} = \int \langle g_{ref}(12) f_{KL}(12) \rangle_{\Omega_1\Omega_2}\, d\mathbf{r}_{12}. \tag{6}$$

Here ρ is the number density of the system, $g_{ref}(12)$ is the pair distribution function of the reference system, $f_{KL}(12)$ is the Mayer function for the site-site square-well potential, i.e $f_{KL}(12) = \exp\left[-\beta U_{KL}(12)\right] - 1$, and $\langle \ldots \rangle_{\Omega_1\Omega_2}$ denotes the angular averaging with respect to the orientations of particles 1 and 2. This integral can be evaluated by choosing an arbitrary location for the origin of the coordinate system associated with each particle. If the origin is placed at the position of the corresponding attractive site of the particle (site K of particle 1 and site L of particle 2), we obtain

$$I_{KL} = 4\pi \int \langle g_{ref}(12) \rangle_{\Omega_1\Omega_2} r_{12}^2 f_{KL}(r_{12})\, dr_{12}, \tag{7}$$

where r_{12} is the distance between sites K and L of particles 1 and 2, respectively, and $\langle g_{ref}(12) \rangle_{\Omega_1\Omega_2}$ is the site-site pair distribution function between two auxiliary sites K and L of the reference system.[S5,S6] For the model at hand the displacement of the sites from the hard-sphere center d is the same for each site, therefore the corresponding site-site distribution function, which we will denote as $g_{ss}^{(ref)}(r)$, does not depend on the type of sites, i.e. $\langle g_{ref}(12) \rangle_{\Omega_1\Omega_2} = g_{KL}^{(ref)}(r) = g_{ss}^{(ref)}(r)$. This correlation function can be calculated either using the reference interaction site model (RISM) approach due to Chandler[S7,S8] or performing direct averaging using the appropriate expression for the hard-sphere radial distribution function. For the model at hand RISM approach is represented by the site-site

Ornstein-Zernike (SSOZ) equation

$$\hat{\mathbf{h}}(k) = \hat{\mathbf{S}}(k)\hat{\mathbf{c}}(k)\hat{\mathbf{S}}(k) + \rho\hat{\mathbf{S}}(k)\hat{\mathbf{c}}(k)\hat{\mathbf{h}}(k), \tag{8}$$

and a Percus-Yevick-like closure relation

$$\begin{cases} c_{\alpha\beta}(r) = \quad 0, & r > \sigma - d\Delta_{\alpha\beta} \\ h_{\alpha\beta}(r) = -1, & r \leq \sigma - d\Delta_{\alpha\beta} \end{cases}, \tag{9}$$

where $\Delta_{\alpha\beta} = 2\delta_{\alpha s}\delta_{\beta s} + \delta_{\alpha 0}\delta_{\beta s} + \delta_{\alpha s}\delta_{\beta 0}$, α and β take the values 0 and s where 0 denotes the center of the particle and s its off-center site, $\hat{\mathbf{S}}(k)$ is a matrix with elements $S_{\alpha\beta}(k) = \delta_{\alpha\beta} + (1 - \delta_{\alpha\beta})\sin(kd)/(kd)$, $\hat{\mathbf{h}}(k)$ and $\hat{\mathbf{c}}(k)$ are matrices with elements $\hat{h}_{\alpha\beta}(k)$ and $\hat{c}_{\alpha\beta}(k)$, which are Fourier transforms of the total and direct site-site correlation functions $h_{\alpha\beta}(r)$ and $c_{\alpha\beta}(r)$, respectively, and $\delta_{\alpha\beta}$ is the Kroneker delta. Alternatively we have[S7,S9]

$$g_{ss}^{(ref)}(r) = \frac{1}{4d^2 r}\int_{|r-d|}^{r+d} dt \int_{|t-d|}^{t+d} \upsilon g_{hs}(\upsilon)\, d\upsilon, \tag{10}$$

where $g_{hs}(r)$ is the radial distribution function of hard spheres. Using the Percus-Yevick expression for $g_{hs}(r)$[S10] , we have

$$g_{ss}^{(ref)}(r) = \frac{\sigma^3}{4d^2 r}\sum_{i=0}^{2}\frac{a_i}{t_i}\left[\frac{1}{t_i}\left(e^{r_d t_i} - 1\right) - r_d\right], \tag{11}$$

where $r \leq 2(\sigma - d)$, $r_d = (r + 2d - \sigma)/\sigma$ and $a_i = t_i L(t_i)/S_1(t_i)$,

$$t_i = \frac{-2\eta + (y_+ j^i + y_- j^{-i})\sqrt[3]{2\eta\xi}}{1-\eta}, \tag{12}$$

$$y_\pm = \sqrt[3]{1 \pm \sqrt{1 + 2\left(\eta^2/\xi\right)^2}}, \tag{13}$$

$\xi = 3 + 3\eta - \eta^2$, $S_1(t) = 3(1-\eta)^2 t^2 + 12\eta(1-\eta)t + 18\eta^2$, $L(t) = (1 + \eta/2)t + 1 + 2\eta$,

$j = \exp\left(2\pi\sqrt{-1}/3\right)$. The corresponding expression for the integral I_{KL} is

$$I_{KL} = \left(e^{-\beta\epsilon_{KL}} - 1\right)\Delta V_{PY}, \tag{14}$$

where

$$\Delta V_{PY} = \frac{\pi}{6}\sum_{i=0}^{2}\frac{a_i}{t_i^4 d^2}\left[6\left(\delta t_i - \sigma\right)e^{\frac{2d+\delta}{\sigma}-1} - \frac{1}{\sigma^2}\sum_{l=0}^{3}P_l t_i^l\right], \tag{15}$$

$P_3 = -8d^3 + 12d^2\sigma + 6(\delta^2 - \sigma^2)d + (2\delta - 3\sigma)\delta^2 + \sigma^3$, $P_2 = 3(-4d^2\sigma + 4d\sigma^2 + \delta^2\sigma - \sigma^3)$, $P_1 = 6\sigma^2(\sigma - 2d)$, $P_0 = -6\sigma^3$. As expected, expression (14) is identical to the corresponding

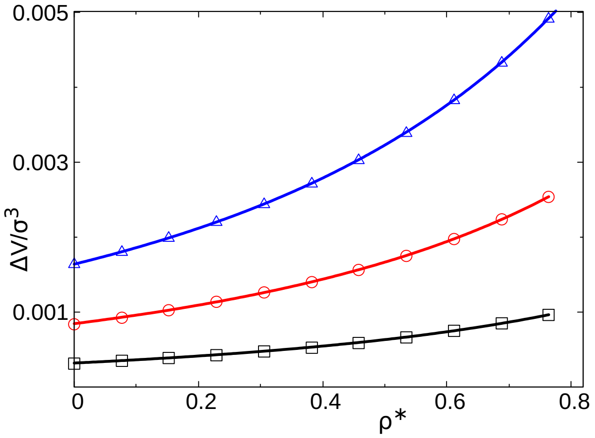

Figure S1: ΔV_{PY} (lines) and $\Delta V_{RISM-PY}$ (symbols) vs density ρ^* at $d = 0.5\sigma$ (black line and squares), $d = 0.45\sigma$ (red line and circles) and $d = 0.4\sigma$ (blue lines and triangles)

.

formula for I_{KL} obtained within Wertheim's original formulation,[S11] in which the origin of the particle coordinate system is located at the hard-sphere center. In figure S1 we compare our results for ΔV_{PY} (15) and $\Delta V_{RISM-PY}$ as a function of the density at three different values of d, i.e. $d = 0.5,\ 0.45,\ 0.4$. Here $\Delta V_{RISM-PY}$ is obtained using $g_{ss}(r)$ calculated by the numerical solution of the RISM equation (8) with PY-like closure relations (9). Excellent agreement is observed, i.e. on the scale of the figure the results for ΔV_{PY} and $\Delta V_{RISM-PY}$ coincide. Thus, for models with a hard-sphere reference system, either of the two methods yields practically identical results. In this case, Wertheim's scheme has the advantage of being simpler and more convenient to apply. However, for models with non-spherical

particles, the evaluation of the key integral I_{KL} within this scheme becomes a formidable task. In contrast, this integral can be computed much more easily using a method based on site–site distribution functions, particularly when the structure of the reference system is described within the interaction site formalism of Chandler et al.[S7] Beyond simple molecular fluids,[S8,S12,S13] models of this type have also been widely used to describe macromolecular and colloidal systems.[S14–S18]

References

(S1) Wertheim, M. S. Fluids with highly directional attractive forces. III. Multiple attraction sites. *J. Stat. Phys.* **1986**, *42*, 459–476.

(S2) Wertheim, M. S. Fluids with highly directional attractive forces. IV. Equilibrium polymerization. *J. Stat. Phys.* **1986**, *42*, 477–492.

(S3) Wertheim, M. S. Thermodynamic perturbation theory of polymerization. *J. Chem. Phys.* **1987**, *87*, 7323–7331.

(S4) Chapman, W. G.; Jackson, G.; Gubbins, K. E. Phase equilibria of associating fluids: chain molecules with multiple bonding sites. *Mol. Phys.* **1988**, *65*, 1057–1079.

(S5) Chandler, D. Derivation of an integral equation for pair correlation functions in molecular fluids. *J. Chem. Phys.* **1973**, *59*, 2742–2746.

(S6) Cummings, P.; Gray, C.; Sullivan, D. Auxiliary sites in the RISM approximation for molecular fluids. *Journal of Physics A: Mathematical and General* **1981**, *14*, 1483.

(S7) Chandler, D.; Andersen, H. C. Optimized cluster expansions for classical fluids. II. Theory of molecular liquids. *The J. Chem. Phys.* **1972**, *57*, 1930–1937.

(S8) Monson, P.; Morriss, G. Recent progress in the statistical mechanical mechanics of interaction site fluids. *Advances in chemical physics* **1990**, *77*, 451–550.

(S9) Holovko, M.; Kalyuzhny, Y. V.; Heinzinger, K. Electrostatic and packing contributions to the structure of water and aqueous electrolyte solutions. *Zeitschrift fur Naturforschung A* **1990**, *45*, 687–694.

(S10) Smith, W.; Henderson, D. Analytical representation of the Percus-Yevick hard-sphere radial distribution function. *Mol. Phys.* **1970**, *19*, 411–415.

(S11) Wertheim, M. Fluids of dimerizing hard spheres, and fluid mixtures of hard spheres and dispheres. *J. Chem. Phys.* **1986**, *85*, 2929–2936.

(S12) Hansen, J.-P.; McDonald, I. R. *Theory of simple liquids: with applications to soft matter*; Academic press, 2013.

(S13) Gray, C. G.; Gubbins, K. E. *Theory of Molecular Fluids: Volume 1: Fundamentals*; Oxford University Press, 1984; Vol. 1.

(S14) Zhang,; Horsch, M. A.; Lamm, M. H.; Glotzer, S. C. Tethered nano building blocks: Toward a conceptual framework for nanoparticle self-assembly. *Nano Lett.* **2003**, *3*, 1341–1346.

(S15) Zhang, Z.; Glotzer, S. C. Self-assembly of patchy particles. *Nano Lett.* **2004**, *4*, 1407–1413.

(S16) Costa, D.; Hansen, J.-P.; Harnau, L. Structure and equation of state of interaction site models for disc-shaped lamellar colloids. *Mol. Phys.* **2005**, *103*, 1917–1927.

(S17) Glotzer, S. C.; Solomon, M. J. Anisotropy of building blocks and their assembly into complex structures. *Nat. Mater.* **2007**, *6*, 557–562.

(S18) Delhorme, M.; Jönsson, B.; Labbez, C. Monte Carlo simulations of a clay inspired model suspension: the role of rim charge. *Soft Matter* **2012**, *8*, 9691–9704.
